# Enhanced oxidative stress resistance in *Ustilago maydis* and its implications on the virulence

**DOI:** 10.1007/s10123-024-00489-8

**Published:** 2024-02-24

**Authors:** Jorge Cuamatzi-Flores, Maritrini Colón-González, Fernanda Requena-Romo, Samuel Quiñones-Galeana, José Antonio Cervantes-Chávez, Lucia Morales

**Affiliations:** 1 Unit for Basic and Applied Microbiology, Faculty of Natural Sciences, Autonomous University of Queretaro, 76230 Querétaro, México; 2 Laboratorio Internacional de Investigación sobre el Genoma Humano, Universidad Nacional Autónoma de México, 76230 Querétaro, México; 3 Escuela Nacional de Estudios Superiores Unidad Juriquilla, Universidad Nacional Autónoma de México, 76230 Querétaro, México

**Keywords:** Fungal pathogens, Adaptation, Oxidative stress, Fungal virulence

## Abstract

The phytopathogenic fungus *Ustilago maydis* causes corn smut by suppressing host plant defenses, including the oxidative burst response. While many studies have investigated how *U. maydis* responds to oxidative stress during infection, the consequences of heightened resistance to oxidative stress on virulence remain understudied. This study aimed to identify the effects on virulence in *U. maydis* strains exhibiting enhanced resistance to hydrogen peroxide (H_2_O_2_).

To achieve this, we exposed *U. maydis* SG200 to 20 escalating H_2_O_2_ shocks, resulting in an adapted strain resistant to concentrations as high as 60 mM of H_2_O_2_, a lethal dose for the initial strain. Genetic analysis of the adapted strain revealed five nucleotide substitutions, two minor copy number variants, and a large amplification event on chromosome nine (1–149 kb) encompassing the sole catalase gene. Overexpressing catalase increased resistance to H_2_O_2_; however, this resistance was lower than that observed in the adapted strain. Additionally, virulence was reduced in both strains with enhanced H_2_O_2_ resistance.

In summary, enhanced H_2_O_2_ resistance, achieved through either continuous exposure to the oxidative agent or through catalase overexpression, decreased virulence. This suggests that the response to the oxidative stress burst in *U. maydis* is optimal and that increasing the resistance to H_2_O_2_ does not translate into increased virulence. These findings illuminate the intricate relationship between oxidative stress resistance and virulence in *U. maydis*, offering insights into its infection mechanisms.

**Supplementary Information:**

The online version contains supplementary material available at 10.1007/s10123-024-00489-8.

## Introduction

Fungal plant pathogens are widespread and exert substantial impacts on crop yield and quality (Avery et al. 2019). Among these pathogens is *Ustilago maydis,* a biotrophic fungus widespread in the Americas, Asia, and Europe, responsible for corn smut disease in *Zea mays*. *U. maydis* has served as an invaluable model organism for investigating the cellular physiology and molecular biology of host–pathogen interactions (Holliday 1961; Schuster et al. 2016; Olicón-Hernández et al. 2019; Dutheil et al. 2020; Lin et al. 2021; López-Martínez et al. 2022; Villagrán et al. 2023), and more recently, the evolutionary history of crop infecting fungi through population genomics (Munkacsi et al. 2006, 2008; Depotter et al. 2021; Schweizer et al. 2021; Momeni and Nazari 2022). The current reference genome of *U. maydis* spans 20.5 Mb and includes 23 nuclear chromosomes, one mitochondrial chromosome, and three unmapped contigs (Kahmann and Kämper 2004; Kämper et al. 2006). Within the annotation of this reference, there are 6,902 protein-encoding genes and two putative functional mobile elements: the retrotransposons *TigR* and *HobS*. This last mobile element consistently appears in either complete or incomplete form, with one copy per chromosome. Mobile elements are commonly found in fungal pathogen genomes and play critical roles in their adaptive processes (Möller and Stukenbrock 2017; Tralamazza et al. 2023). These elements have been associated with various chromosomal rearrangements including amplifications, deletions, translocations, and inversions, in yeasts and other fungi (Daboussi 1996; Wang et al. 2020a, b).

Fungal pathogens have a remarkable ability to adapt to their hosts and to changing environments influenced by drugs or stressors (Stukenbrock and Croll 2014; Grandaubert et al. 2019; Habig et al. 2021; Möller et al. 2021). These adaptations frequently encompass significant changes at the chromosomal level, including gains or losses of entire chromosomes or a fraction of them. They may also entail epigenetic modifications affecting regulation of gene expression and alterations in mutation rate (Selmecki et al. 2009; Gilchrist and Stelkens 2019; Kramer et al. 2023; Vande Zande et al. 2023).

While there has been extensive research on *U. maydis* and its infection process in maize, the consequences of increasing oxidative stress resistance on virulence remain elusive. Notably, specific gene deletions have been shown to decrease both virulence and the ability to withstand oxidative stress. For instance, disruptions in *tps2*, a gene involved in trehalose biosynthesis (Cervantes-Chávez et al. 2016), and deletions of the *yap1* transcription factor, which plays a role in oxidative stress response (Molina and Kahmann 2007), have been identified. While numerous studies have explored the impact of specific genes on pathogenicity, it remains unclear whether increased resistance to oxidative stress directly translates into enhanced virulence.

This study investigates the adaptive response of *U. maydis* to oxidative stress and its implications in virulence. We used as the initial strain the haploid and solopathogenic *U. maydis* SG200 (Kämper et al. 2006), which is capable of infecting without mating, enabling subsequent experiments in maize plants. We exposed this initial strain to periodic H_2_O_2_ shocks over 200 generations, resulting in significant H_2_O_2_ adaptation. In this study, we compared the virulence between SG200, a H_2_O_2_-adapted strain (UmH_2_O_2_-R) and a strain with an extra catalase copy (oexUMAG_11067).

## Results

### Hydrogen peroxide adaptation and inheritance of H_2_O_2_-resistance in *Ustilago maydis*

Adaptation is a biological process through which organisms gradually acquire physiological and genetic changes over successive generations, enhancing their fitness. This enhanced fitness is reflected in their capacity to thrive and propagate in specific environmental conditions. In this study, we took advantage of the adaptive potential of *U. maydis* SG200 to obtain the strain UmH_2_O_2_-R, showing increased resistance to H_2_O_2_. This strain was obtained by selecting surviving cells after periodic exposures to increasing concentrations of H_2_O_2_, starting at 5 mM and reaching 60 mM after twenty treatments.

**Fig. 1:**
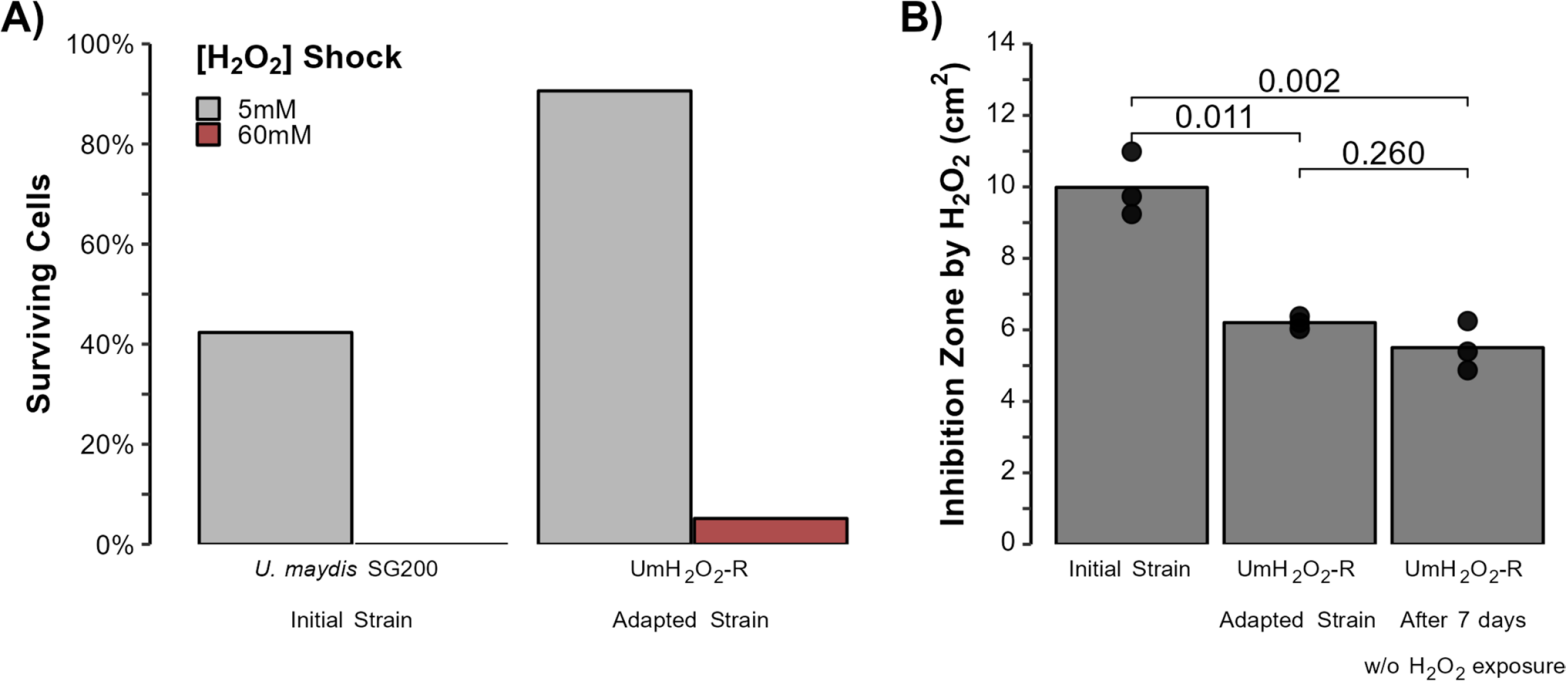
Hydrogen Peroxide Resistance in *Ustilago maydis*. **A** Percentage of cells surviving H_2_O_2_ shocks at 5 and 60 mM in the initial strain *U. maydis* SG200 and the adapted strain (UmH_2_O_2_-R). **B** Inhibition zone areas produced by H_2_O_2_ of the initial strain and of UmH_2_O_2_-R immediately after isolation, and after 7 days of culture with daily dilutions in the absence of H_2_O_2_ (UmH_2_O_2_-R + 7 days)

Adaptation to H_2_O_2_ increased by the end of the 20 treatments. As depicted in Fig. 1A, we assessed the percentage of viable cells by quantifying colony-forming units (CFUs) following a three-hour exposure to 5 and 60 mM H_2_O_2_, using the number of CFUs without exposure as baseline. At a 5 mM concentration, the survival rate increased significantly from 42% in the initial strain, to 91% in the adapted strain UmH_2_O_2_-R. A 95% confidence interval (32.39033, 52.34300) was calculated for the survival percentage at 5 mM for the initial strain. Notably, the measurement obtained for the adapted strain after a shock at the same concentration lies outside of this confidence interval. Exposure to 60 mM H_2_O_2_ shock was lethal for the initial strain however, we observed a survival rate of 5% in the evolved strain UmH_2_O_2_-R. These observations indicate that periodic exposure to H_2_O_2_ significantly improved the resistance of *U. maydis* to this oxidative agent. The adapted strain, UmH_2_O_2_-R, even survived a shock with a concentration that was previously lethal for the initial strain.

To assess the stability of H_2_O_2_-resistance phenotype, UmH_2_O_2_-R was cultured for seven days with daily dilutions in the absence of H_2_O_2_. We evaluated the H_2_O_2_ resistance by measuring the area of inhibition in an agar diffusion assay. Figure 1B shows that there was no significant difference in the inhibition zone between UmH_2_O_2_-R right after the twentieth treatment and following its propagation for seven days without H_2_O_2_ (as determined by paired *t-*test, *p*-value = 0.260). Furthermore, in both scenarios H_2_O_2_ inhibition was lower than in the initial strain *U. maydis* SG200 (with *p*-values of 0.011 and 0.002, respectively).

### Genomic variants found after hydrogen peroxide adaptation in *Ustilago maydis*

Due to the hereditary nature of the resistance phenotype, we aimed to identify the genetic variants that accumulated in the short adaptation period. We conducted whole-genome sequencing on both the initial strain (SG200) and the H_2_O_2_-adapted strain (UmH_2_O_2_-R). After a rigorous filtering we identified five *bona fide* single nucleotide variants (SNVs) distributed throughout the nuclear and mitochondrial genomes of the adapted strain (Supplementary Table 1). Three SNVs were non-synonymous substitutions, while the other two SNVs were present in intergenic regions.

The first non-synonymous substitution was located at position Chr01:2,430,690, within the UMAG_00813 gene (Chr01:2,430,525–2,432,966 ( +)). This gene encodes a putative protein containing the FAD/NAD(P)-binding domain, as predicted by AlphaFold2 (reference entry: A0A0D1E974). This mutation resulted in an amino acid substitution, specifically changing glycine to arginine at position 56 (Gly56Arg).

The second non-synonymous substitution was found at Chr08:464,088, within the UMAG_10823 gene (Chr08:463,697 – 464,216 (-)). This gene encodes a putative long chronological lifespan protein, as predicted by AlphaFold2 (reference entry: A0A0D1C4W6). The mutation led to an amino acid substitution, replacing glutamic acid with aspartic acid at position 43 (Glu43Asp).

The last non-synonymous substitution occurred at Chr18:330,272 within the UMAG_05545 gene (Chr18:328,759 – 331,155 ( +)). This gene encodes a putative histone-lysine N-methyltransferase with a Dot1 domain responsible for histone H3 lysine 79 (Lys-79) methylation. AlphaFold2 (reference entry: Q4P2W8) predicted this function. Specifically, the nucleotide substitution involved a C to A transversion at position 330,272, resulting in the replacement of the threonine residue with an asparagine residue (Thr505Asn) within the Dot1 domain, a critical region for the catalytic activity of the protein (Lee et al. 2018; Min et al. 2003).

The intergenic substitutions were a T to A transversion located at Chr10:623,352( +), and a G to A transition in the mitochondrial genome at position 15,246( +).

In addition to single nucleotide variants, copy number variants (CNVs) can be induced in response to environmental stressors, conferring rapid adaptive advantages. We found three CNVs that were only present in the adapted strain (Supplementary Table 2). Among these, two were relatively small, with less than 5 kb, while the third CNV was substantially larger, spanning approximately 150 kb.

One of the CNVs was situated on chromosome twenty from base 50,551 to base 51,600 (1,050 bp), exhibiting a copy number of 3.74. Although this sequence lacks annotated genes, it contains a 127 bp segment that is recurrently found throughout the genome. The second CNV was identified on chromosome seventeen from position 151 to 4,650 (4,500 bp), with a copy number of 1.80. This sequence overlaps with the UMAG_04693 gene (Chr17:1396–4642 ( +)), which encodes a putative protein containing a helicase ATP-binding domain.

The third CNV, identified by CNVnator ranged from position 1 to 149,100 of chromosome nine, and displayed a copy number of 2.99 (as illustrated in Supplementary Fig. 1A). This CNV presented a log_2_ ratio of 1.54 in the normalized coverage, signifying a total copy number of three for the corresponding sequence, consistent with CNVnator’s results. In this 150 kb region, we identified a total of 55 protein-coding genes, which are listed in Supplementary Table 3. Among these genes, UMAG_11067 (Chr9:18,661–20,910 (-)) stood out as the sole catalase enzyme within the *U. maydis* reference genome. This enzyme plays a critical role in the breakdown of hydrogen peroxide into water and oxygen, making it a promising candidate for studying the impact of its duplication on resistance to H_2_O_2_ and virulence.

To explore the breakpoint of the CNV on chromosome 9 in greater detail, we conducted an in-depth analysis within the amplified region, at one bp resolution. The end of the amplification found by CNVnator (Chr9:149,100) falls within a 4.5 kb region lacking annotated protein-coding genes. Nevertheless, within the region spanning positions 147 kb to 150 kb, we observed a slight decrease in the log_2_ ratio of normalized coverage, as visualized in Supplementary Fig. 1B. Inside this region, we also identified two remnants of the 750 bp-HobS direct repeats, oriented in an inverted direction at positions Chr9:147,098–147,496 and Chr9:149,294–148,883. Two fragments of 400 bp and 412 bp of these remnants align with the HobS direct repeats with 91% and 92% identity, respectively; they may be responsible for the genomic instability of the region and its triplication.

### Triplication of the catalase gene enhances expression in response to hydrogen peroxide in the adapted strain

**Fig. 2:**
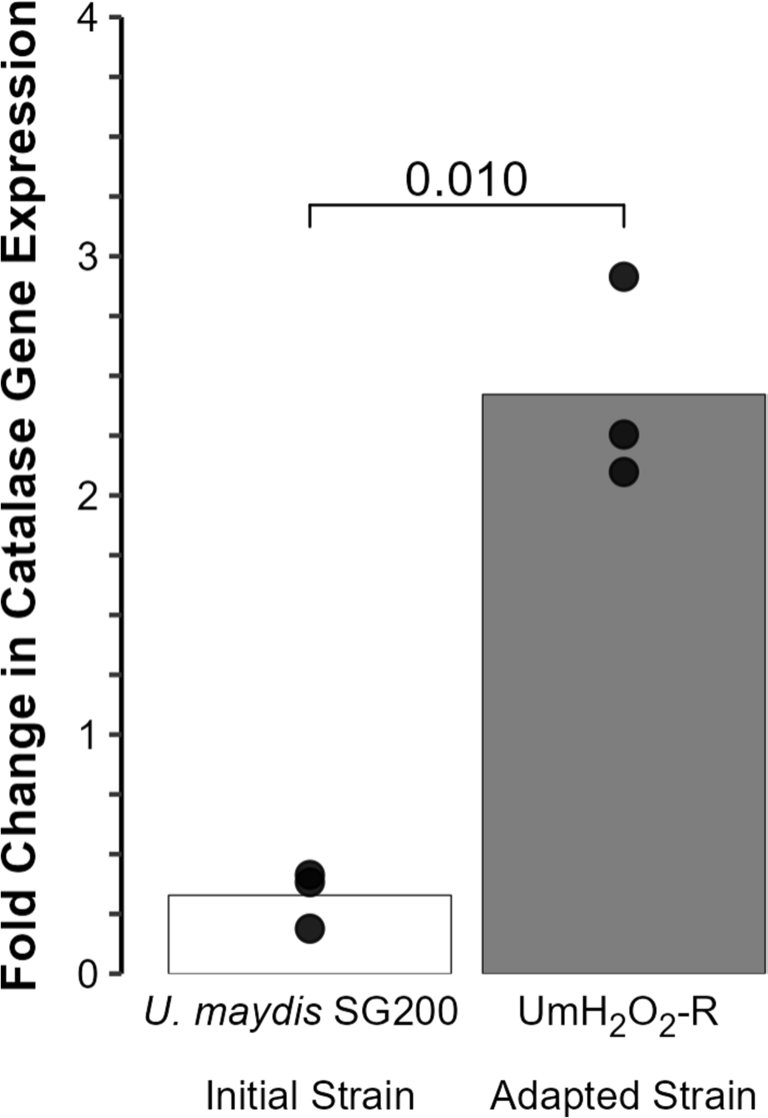
Changes in catalase gene expression. Fold change gene expression of UMAG_11067 (catalase encoding gene) following an exposure at 10 mM of H_2_O_2_

To determine whether the observed increase in the copy number of the catalase gene in the adapted strain UmH_2_O_2_-R resulted in elevated transcript levels, we conducted a gene expression analysis using qPCR. After a 10 mM H_2_O_2_ shock, we observed a 2.1-fold increase in catalase gene expression within the strain bearing the triplication compared to the initial strain *U. maydis* SG200. This difference in expression levels was found to be statistically significant with a *p-*value of 0.010 as determined by *t*-test (Fig. 2).

### Increased catalase expression enhances hydrogen peroxide resistance but diminishes virulence in maize

**Fig. 3:**
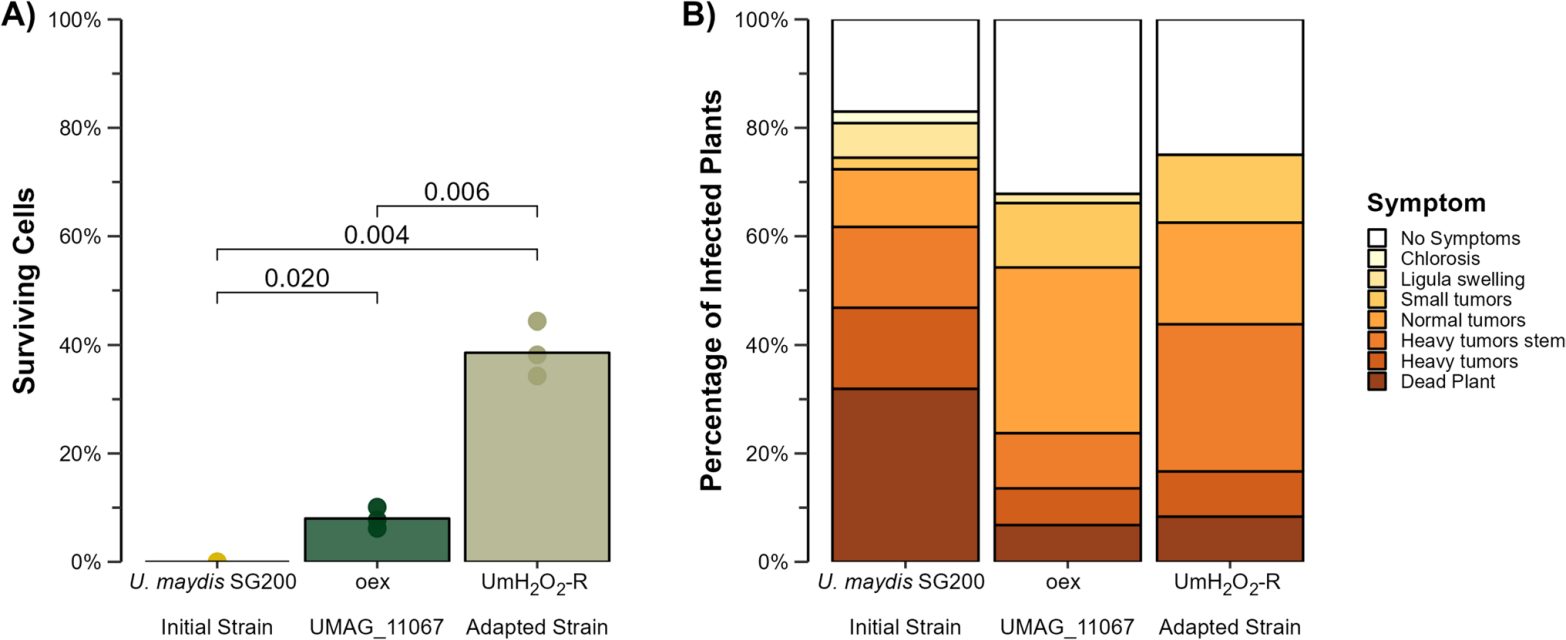
Hydrogen peroxide resistance and virulence profiles of oexUMAG_11067 and UmH_2_O_2_-R. **A** Percentage of viable cells determined through CFU counts after exposure to 10 mM H_2_O_2_ shock. **B** Virulence assessment on maize seedlings analyzed at 12 days post infection. Color coding represents the severity of infection

To test the potential role of the catalase gene in enhancing H_2_O_2_ resistance, we engineered an overexpression strain carrying an additional copy of UMAG_11067 (oexUMAG_11067). Both the oexUMAG_11067 and UmH_2_O_2_-R strains exhibited significant differences in the survival rates following exposure to the 10 mM H_2_O_2_ shock, compared to *U. maydis* SG200 (*t*-test *p*-value = 0.020 for oexUMAG_11067, and *p*-value = 0.004 for UmH_2_O_2_-R), where this concentration proved completely inhibitory (Fig. 3A). Specifically, the oexUMAG_11067 strain displayed an 8% survival rate, whereas survival rate in UmH_2_O_2_-R was 38.6%. These results confirmed that catalase overexpression enhanced resistance to H_2_O_2_. However, it is important to note that despite this enhancement, the overexpression mutant failed to achieve the same level of resistance observed in the adapted strain UmH_2_O_2_-R.

To evaluate the virulence of *U. maydis* SG200, UmH_2_O_2_-R, and oexUMAG_11067, we monitored symptoms in maize seedlings at 12 days post-infection. Figure 3B illustrates that the two strains exhibiting increased H_2_O_2_ resistance, specifically oexUMAG_11067 and UmH_2_O_2_-R, elicited milder symptoms when compared to the *U. maydis* SG200 strain (chi-square, *p-*value = 2.64 × 10^–4^ for oexUMAG_11067 and chi-square, *p-*value = 4.8 × 10^–3^ for UmH_2_O_2_-R). The most significant difference was the reduction of deceased maize plants. Furthermore, we observed an increase in the proportion of symptom-free plants and a decrease in the number of individuals displaying severe tumors (Supplementary Fig. 2). Moreover, the disparities in symptoms between oexUMAG_11067 and UmH_2_O_2_-R were not statistically significant (chi-square, p-value = 0.299). These collective findings conclusively indicate that enhanced resistance to hydrogen peroxide, whether achieved through catalase overexpression (oexUMAG_11067) or adaptive exposure (UmH_2_O_2_-R), did not translate into increased virulence on maize.

## Discussion

The main goal of this study was to enhance H_2_O_2_ resistance in *Ustilago maydis* SG200 strain, identify the genetic changes occurring during adaptation, and subsequently evaluate the impact of the enhanced H_2_O_2_ resistance on virulence in maize.

Adaptation occurs when individuals of a species increase their fitness within a specific environment. In this work we induced oxidative stress using H_2_O_2_, a highly reactive molecule that at high concentrations, can cause damage to various cellular components, ultimately leading to cell death. Despite being lethal at high concentrations, exposure to milder concentrations of this reagent can lead to adaptation in different fungal species (Linder et al. 2017; Huang and Kao 2018; Qi et al. 2019; Zhang et al. 2019). In our study, we observed that the adapted UmH_2_O_2_-R strain exhibited increased survival rate when exposed to H_2_O_2_ concentrations that were lethal to the *U. maydis* SG200. These findings align with research conducted on the pathogenic yeast *Candida glabrata* (Huang and Kao 2018). In *C. glabrata*, an increase in H_2_O_2_ resistance was observed when the organism was subjected to 22 periodic H_2_O_2_ shocks, starting from an initial concentration of 80 mM and reaching 350 mM by the end of the experiment. Both our observations in *U. maydis* and the results in *C. glabrata* (Huang and Kao 2018) evidence the capacity of fungal species to rapidly adapt to oxidative stress induced by H_2_O_2_. In both cases, within just 180 generations for *C. glabrata,* and 200 generations for *U. maydis*, both species were able to survive to a previously lethal concentration of H_2_O_2_.

Then, we analyzed the genomes of the initial strain and the adapted strain, UmH_2_O_2_-R. Variants already present in the initial strain were considered as the genetic background and were removed from the set of identified variants in UmH_2_O_2_-R. Regarding single nucleotide variants, we identified two intergenic mutations and three non-synonymous mutations. One intergenic SNV was located within the promoter region of the gene UMAG_03851, a putative adenylosuccinate synthase. The other intergenic SNV was found in the mitochondrial genome (Mt:15,246), with no annotated genes in the 2 kb periphery.

The non-synonymous nucleotide substitutions were detected in three distinct genes: UMAG_00813, which encodes a putative protein containing the FAD/NAD(P)-binding domain; UMAG_10823, coding for a putative long chronological lifespan protein; and UMAG_05545, encoding a histone-lysine *N*-methyltransferase (HKMTs) with a Dot1 domain. These functional annotations were all predicted by AlphaFold2 (Jumper et al. 2021), nevertheless the prediction confidence is low for UMAG_00813 and UMAG_10823, but more reliable for UMAG_05545.

The UMAG_00813 protein with a FAD/NAD(P)-binding domain, falls under the gene ontology category of oxidoreductase activity, suggesting a connection to oxidative stress. In the adapted strain, UmH_2_O_2_-R, the identified mutation occurred in the protein’s amino-terminal region.

In the case of UMAG_10823, the amino acid substitution occurred within the signal peptide. Although AlphaFold2 prediction for UMAG_10823 as a putative long chronological lifespan protein has a low confidence, previous research has observed an association between antioxidant defense and extended long chronological lifespan in the yeast *S. cerevisiae* (Mirisola and Longo 2022).

The last non-synonymous substitution occurred within the catalytic domain of the protein encoded by UMAG_05545, which is a putative histone-lysine *N*-methyltransferase with a Dot1 domain. This protein is also known as Dot1 and catalyzes the methylation of lysine 79 on histone H3 (Ng et al. 2002). Histone methylation plays a critical role in the regulation of transcription and the structure of chromatin (Brosch et al. 2008; Li et al. 2019). Disrupting the catalytic activity of Dot1 leads to the repression of telomeric silencing in both *S. cerevisiae* and *Penicillium oxalicum* (Ng et al. 2002; Li et al. 2019). In *Aspergillus flavus* deletion of *dot1* reduces the virulence on maize seeds and increases resistance to oxidative stress induced by *tert*-butyl hydroperoxide (Liang et al. 2017). Furthermore, previous studies have reported that the inactivation of genes associated with chromatin remodeling reduces the virulence of fungal plant pathogens (Gu et al. 2017; Wang et al. 2020a, b). The mutation within the Dot1 domain of the protein encoded by UMAG_05545 could potentially impact the activity of this protein and consequently have an impact in the chromatin structure of *U. maydis*.

In addition to single nucleotide mutations, we identified copy number variants upon H_2_O_2_ exposure in the UmH_2_O_2_-R strain. In mammalian cells, abnormal chromosome numbers or large chromosomal duplications often have deleterious effects (Tosh et al. 2022; Williams et al. 2008). However, in the case of filamentous fungi and yeast, chromosomal duplications play a key role in facilitating rapid adaptation to stressful environments by increasing the copy number of genes associated with stress response (Selmecki et al. 2009; Chen et al. 2012; Linder et al. 2017; Gilchrist and Stelkens 2019; Khateb et al. 2023).

The presence of catalase UMAG_11067 within the amplified segment of chromosome 9, along with its elevated gene expression, suggests a potential role in the observed H_2_O_2_ resistance in UmH_2_O_2_-R. Our findings regarding the increased copy number of catalase in *U. maydis* is analogous to observations in *S. cerevisiae*. In this yeast, partial chromosomal amplifications harboring H_2_O_2_-degrading proteins, such as cytoplasmic thioredoxin peroxidase (*tsa2*) and cytosolic catalase (*ctt1*), have been reported in response to oxidative stress induced by H_2_O_2_ (Linder et al. 2017; Zhang et al. 2019). The contribution of catalase to H_2_O_2_ resistance was evaluated by overexpressing this gene in the oexUMAG_11067 strain. While oexUMAG_11067 showed increased resistance, it was not as pronounced as the one achieved through adaptive evolution in the UmH_2_O_2_-R strain, suggesting that the other identified genetic variants, or any other epigenetic mechanisms that arose during this process may also play a role.

In the final stage of our research, we evaluated the consequences of enhanced H_2_O_2_ resistance on the pathogenicity of *U. maydis* in maize seedlings. Our findings indicate that both the UmH_2_O_2_-R and oexUMAG_11067 strains of *U. maydis* show a decrease in virulence. The common genetic feature in these strains, not present in the initial strain SG200, is an increased number of catalase gene copies. This suggests a correlation between the overexpression of catalase and the observed reduction in virulence, a phenomenon also present in *Candida albicans* where the demand for iron by catalase—a cofactor scarce in host tissues—has been linked to decreased virulence (Pradhan et al. 2017). Both *C. albicans* and *U. maydis* rely on iron as a catalase cofactor. Additionally, the research by Eichhorn et al. (2006) supports the need of iron for virulence, showing that *U. maydis* strains with compromised iron uptake are less virulent.

If iron levels are low, the metabolic expense of catalase production becomes detrimental, as the enzyme will be non-functional without its cofactor. Moreover, catalase intense iron requirement would reduce its availability for other essential metabolic processes such as DNA synthesis and respiration. While we lack specific data on the iron content in maize plants and whether it meets the requirements of strains with high catalase expression, increasing iron content in the plant might potentially reverse the reduced virulence. Nonetheless, it is still an open question whether *U. maydis* can efficiently use iron from supplemented maize. It is important to consider that supplying external iron can suppress the expression of the entire iron-related gene cluster (Eichhorn et al. 2006).

Our research evidenced *U. maydis* capacity for rapid adaption to H_2_O_2_ and the genetic variants linked to this process. To uncover the potential roles of the identified genetic variants, and of any potential epigenetic modifications that may have emerged during the adaptation process further investigation would be required.

In conclusion, our study presents a strategy for selecting fungi adapted to harsh environmental conditions. This research also highlights the remarkable adaptive potential of *U. maydis* in response to H_2_O_2_ stress through large chromosomal duplications. Additionally, it illustrates the trade-offs associated with this adaptation, notably the reduction in virulence. These findings enrich our understanding of how fungi rapidly adapt and paves the way for forthcoming research aiming to study the molecular mechanisms involved in these processes.

## Materials and methods

### Cultures and growth conditions

The solopathogenic strain *Ustilago maydis* SG200 (Kämper et al. 2006) was employed for both, H_2_O_2_ adaptation (UmH_2_O_2_-R) and the generation of a strain with catalase overexpression (oexUMAG_11067). Liquid cultures of *U. maydis* were cultivated in CM broth (Holliday 1961) and incubated at 28 °C with continuous agitation at 180 rpm using an orbital shaker. For agar-based assays, CM broth was supplemented with 2% microbiological agar, and the plates were incubated at 28 °C for 48 h.

### Assessment of H_2_O_2_ resistance through area of inhibition in agar diffusion assay

To evaluate resistance to H_2_O_2_, we inoculated standard round petri dishes (CM plates) with a 100 μL solution containing 10^7^ cells/mL, using a sterile L-shaped spreader. Subsequently, we placed a 5 μL droplet of 30% (w/w) H_2_O_2_ (H1009, Sigma Aldrich) on a sterile filter paper disk at the center of the plate. The area of the inhibition halo produced by the H_2_O_2_ droplet was measured. To determine the statistical significance of differences of the inhibition area, a *t-*test was conducted using the rstatix package (Kassambara 2020) within R software, version 4.3.0 (R Development Core Team 2023).

### Generation of the adapted strain UmH_2_O_2_-R

An isogenic culture of *U. maydis* SG200 served as the starting point for generating the adapted strain UmH_2_O_2_-R. This adaptation process encompassed a series of 20 treatments, during which H_2_O_2_ concentrations were incrementally raised, starting at 5 mM and reaching a peak of 60 mM (refer to Supplementary Table 4). Each treatment was divided into two phases: a shock phase and a recovery phase (see Supplementary Fig. 3). In the shock phase, 10^6^ cells of the previous treatment were exposed to H_2_O_2_ for 3 h in 1 mL of CM broth and incubated at 28 °C with continuous agitation at 500 rpm, in a 1.5 mL tube inside a thermoblock (ThermoMixer C, Eppendorf). At the end of the shock phase, we used 0.1 mL to estimate the survival rate of the exposed cells through quantification of CFUs. For this, the cells were diluted 1:1000 and 0.1 mL of that dilution was evenly distributed on CM agar plates. Subsequently, the recovery phase started by transferring the remaining 0.9 mL of the shock-surviving cell population into 19.1 mL of CM broth, which was then incubated at 28 °C with agitation at 180 rpm for 45 h. This entire process was iterated 20 times, with H_2_O_2_ concentrations being raised in incremental steps every two treatments. The H_2_O_2_-adapted strain, UmH_2_O_2_-R was isolated from the shocked cells after the twentieth treatment with H_2_O_2_ at 60 mM. The colony was randomly selected from the plate on which CFUs were quantified. Then it was streaked twice on CM agar to obtain single isolated colonies. One colony from the last plate was randomly chosen and named UmH_2_O_2_-R. Then, UmH_2_O_2_-R was cultured in CM broth for 24 h. A portion of this last culture was cryo-preserved, while the remaining cells were used for DNA extraction and sequencing.

### Inheritance of H_2_O_2_ resistance in the adapted strain UmH_2_O_2_-R

To investigate whether the observed H_2_O_2_ resistance in the adapted strain UmH_2_O_2_-R was a stable inheritable trait, it was propagated for seven days in CM broth without exposure to H_2_O_2_, with daily dilutions. Subsequently, we assessed its resistance using agar diffusion assays, measuring the area of inhibition both immediately after isolation and at the end of the seven-day propagation period without any exposure to H_2_O_2_.

### DNA extraction and sequencing

Genomic DNA extraction was carried out using a mechanical lysis-based protocol using 0.3 mm glass beads (SigmaAldrich, G1277) and phenol:chloroform:isoamyl alcohol (SigmaAldrich, 77,617). DNA concentration was quantified using a fluorometric assay with the Qubit 2.0 Fluorometer (Life Technologies) and the Qubit dsDNA BR Assay kit (ThermoFisher, Q32853). DNA purity was determined by assessing the absorbance ratio at 260/280 nm using a Nanodrop, while DNA integrity was verified through electrophoresis on a 0.8% agarose gel stained with SYBR Safe (ThermoFisher, S33102). Whole genome sequencing, for the initial strain *U. maydis* SG200 and for the adapted strain UmH_2_O_2_-R, was conducted using short-read 150 bp paired-end sequencing on the DNBseq platform at BGI.

### Genome sequence analysis

#### Read preprocessing and alignment

The raw short reads, for initial strain *U. maydis* SG200 and for the adapted strain UmH_2_O_2_-R**,** were preprocessed using fastp v.0.20.0 (Chen et al. 2018) with default parameters. Subsequently, they were aligned to the *U. maydis* 521 v.2 reference genome (available at https://fungi.ensembl.org/Ustilago_maydis/Info/Index, download date: 19–10-2019; last accessed on 22–08-2022) using BWA-MEM v.0.7.8 (Li and Durbin 2010). Duplicated reads were identified and flagged using the picard toolkit v.2.6.0 (Broad Institute 2016). The final alignment outputs were first exported as a bam files and subsequently compressed into cram files.

#### SNV identification

To identify Single Nucleotide Variants (SNVs), we used the cram-formatted alignment files as input for bcftools mpileup v.1.9 (Li et al. 2009). Base quality (-Q) and mapping quality (-q) thresholds were set at 20 and 30, respectively. SNVs were called from the mpileup file using bcftools call with the multiallelic caller option (-m) and specifying ploidy as 1 (–ploidy 1). SNVs coming from the genetic background of SG200, 858 out of 899, were identified and subsequently removed using vcfR v.1.14.0 (Knaus and Grünwald 2017), dplyr v.1.1.2 (Wickham et al. 2023), and data.table v.1.14.8 (Dowle and Srinivasan 2023) packages with a customized R script. Subsequently, the remaining 41 SNVs were further filtered by setting a quality score threshold greater than 200 (Q > 200). We then determined whether the SNVs were located within a gene or were intergenic using the gene annotation file of *U. maydis* v.2, accessible at: https://ftp.ensemblgenomes.ebi.ac.uk/pub/fungi/release-57/gff3/ustilago_maydis, last accessed on 2022–08-22). This annotation was processed with pandas v.2.0.3 (McKinney 2010) in a customized Python script (Python Software Foundation 2016).

#### CNV detection

To detect the CNVs (Copy Number Variants) arising during the generation of the adapted strain UmH_2_O_2_-R, we employed CNVnator, following the methodology outlined by Abyzov et al. (2011), with a bin width of 150 bp. We subsequently removed the CNVs that were already present in the initial SG200 strain, representing the departing genetic background. Additionally, variants with an e-value greater 0.05 were excluded. We also computed the log_2_ ratio of normalized coverage at base-pair resolution to visualize the breakpoints of the large chromosomal amplifications.

For a functional assessment of the genes involved in SNVs or CNVs, we consulted the *U. maydis* UniProt database obtained with AlphaFold2 (https://www.uniprot.org/taxonomy/237631, last accessed 2023/01/20).

### Quantification of catalase gene (UMAG_11067) expression

To assess the expression of UMAG_11067 (catalase) in *U. maydis* strains SG200 and UmH_2_O_2_-R, quantitative polymerase chain reaction (qPCR) was performed on the StepOnePlus™ system (Applied Biosystems™) using Maxima SYBR Green/ROX qPCR Master Mix (2X). The UMAG_04869 gene that encodes the elongation factor subunit 2b (elF2b) served as the reference for normalization. Total RNA was extracted from 10^8^ cells after a three-hour exposure to 0 and 10 mM of H_2_O_2_, following the manufacturer’s instructions for the TRIzol™ reagent. Subsequently, first-strand cDNA synthesis was carried out using SuperScript® III Reverse Transcriptase with 300 ng of total RNA as initial template. The efficiencies of the qPCR reactions for both the target and reference genes were determined through standard curves generated with 1 µg of cDNA. Changes in gene expression were calculated by comparing UMAG_11067 gene expression levels without H_2_O_2_ exposure (0 mM), following the method described by Pfaffl, (2001). Primer sequences are provided in Supplementary Table 5.

### Generation of catalase overexpression strain (oexUMAG_11067)

To create the catalase overexpression strain (oexUMAG_11067), we introduced the open reading frame (ORF) of UMAG_11067 into the *ip locus*, under the regulatory control of the OMA promoter and the NOS terminator. Initially, the UMAG_11067 ORF was amplified via PCR and linked with the NOS terminator, as well as a segment located one kilobase upstream of the *ip locus*. This resulting construct was then inserted into the plasmid pUMa2625 at the *Nco*I – *EcoR*I sites. The recombinant vector obtained was subsequently transformed into *Escherichia coli* by heat shock and selected on LB agar containing 100 μg/mL of ampicillin. *E. coli* clones were subcloned twice on plates with antibiotics and cultured in LB broth supplemented with 100 μg/ml of ampicillin. Plasmid identity was confirmed through restriction analysis using *Nco*I, *Nco*I + *Eco*RI, and *Nco*I + *Sda*I, and validated by Sanger sequencing. The transforming molecule was excised from the resultant plasmid through *Age*I + *EcoR*I digestion and purified from a 0.8% agarose gel.

Protoplasts of *U. maydis* strains were prepared during the mid-exponential growth phase using Lytic enzymes from *Trichoderma harzianum* (SigmaAldrich, L1412). The protoplasts were combined with 3 μg of DNA and selected on media containing 3 μg/mL of carboxin. Transformant clones were streaked three times on antibiotic-containing plates. Subsequently, overexpressing strains were verified through end-point PCR. Oligonucleotide sequences used in generating this strain are provided in Supplementary Table 5.

### Assessment of H_2_O_2_ resistance through CFU counting

To evaluate resistance to H_2_O_2_ in the strains SG200, UmH_2_O_2_-R and oexUMAG_11067, we employed a quantification method based on colony-forming units (CFU) following exposure to a three-hour shock with 10 mM H_2_O_2_. To establish control conditions for CFU comparisons, an equivalent number of cells (10^6^) was exposed to 0 mM H_2_O_2_. After the exposure period, both the shocked and untreated cell populations underwent serial dilution until 10^–3^. Subsequently, 100 µL of each dilution were uniformly spread onto CM agar plates using sterile 0.5 mm-diameter glass beads. These plates were then incubated at 28 °C for 48 h. The percentage of surviving cells after the 10 mM H_2_O_2_ shock treatment was estimated by referencing the CFU counts obtained under 0 mM H_2_O_2_ conditions as a baseline reference. To discern statistical variations in the percentage of surviving cells, a *t*-test was performed using R software v.4.3.0 (R Development Core Team 2023).

### Pathogenicity test

We assessed the virulence symptoms produced by *U. maydis* SG200, *U. maydis* UmH_2_O_2_-R, and *U. maydis* oexCAT on nine-day-old *cacahuazintle* maize seedlings, that were infected with 300 μL of a cellular suspension at 10^8^ cells/mL of each strain injected at the stem. The pots with maize seedlings were maintained in a green-house. Disease symptoms were recorded at 12 days post-infection. Statistical significance was tested with a chi-squared test in R software v.4.3.0 (R Development Core Team 2023). All plots were generated using the package ggplot2 (Wickham 2016).

## Supplementary Information

Below is the link to the electronic supplementary material.

Supplementary file1 (PDF 1279 KB)

Supplementary file2 (XLSX 36.3 KB)

Supplementary file3 (XLSX 12 KB)

Supplementary file4 (PDF 458 KB)

## Data Availability

The code and input files required to replicate the analyses conducted in this study are accessible at: https://github.com/JLuisCuamatzi/USMA_H2O2_Adaptation. The raw reads for *U. maydis* strains SG200 and UmH_2_O_2_-R genomic data can be obtained from the National Center for Biotechnology Information (NCBI) under accession numbers SRR25650020 and SRR25650011, respectively.
