# Supplementary material for: Enhanced oxidative stress resistance in Ustilago maydis and its implications on the virulence
Source: Int Microbiol. 2024 Feb 24;27(5):1501–11. doi: 10.1007/s10123-024-00489-8 (PMC11452521; doi:10.1007/s10123-024-00489-8)
Supplement: Supplementary file 1 — Supplementary file1 (PDF 1279 KB) [file 10123_2024_489_MOESM1_ESM.pdf]

Supplementary Material

Supplementary Table 1. Single Nucleotide Variants detected in the sequenced colony UmH_2O_2.R-Col after adaptation to hydrogen peroxide. [a]Mitochondrial genome.

SNP Location	Gene ID	Protein Name	Nucleotide Substitution	Amino acid Substitution
Chr01:2,430,690	UMAG_00813	FAD/NAD(P)-binding domain-containing protein	G > C	Gly56Arg
Chr08:464,088	UMAG_10823	Long chronological lifespan protein 2	T > A	Glu43Asp
Chr10:623,352	Intergenic	-	T > A	-
Chr18:330,272	UMAG_05545	Histone-lysine N-methyltransferase	C > A	Thr505Asn
Mt[a]:15,246	Intergenic	-	G > A	-

Supplementary Table 4. Hydrogen peroxide concentrations used during adaptation of *U. maydis* SG200.

Treatment	Time (h)	H_2O_2 (mM)
1	48	5
2	96	5
3	144	8
4	192	8
5	240	11
6	288	11
7	336	14
8	384	14
9	432	17
10	480	17
11	528	20
12	576	20
13	624	30
14	672	30
15	720	40
16	768	40
17	816	50
18	864	50
19	912	60
20	960	60

Supplementary Table 5. Oligonucleotides used in this study.

Oligonucleotide name	Sequence (5' – 3')	Template	Product size (bp)	Purpose
Oex-Um11067_1	[1]*tttt***CCATGG**AGTCACTCGGTCAAAATGGGAGAG	*U. maydis* gDNA	2299	To amplify the UMAG_11067 ORF
Oex-Um11067_2	[2]tttgaacgatcGAGCGGAGCATGGTTAGAACC			
Oex-NOSt_1	[2]ATGCTCCGCTCgatcgttcaaacatttggcaataaagtttcttaag	Plasmid pUMa2625	276	To amplify NOS terminator for further fusion with UMAG_11067 ORF
Oex-NOSt_2	[2]GGACAGCACCGCgatctagtaacatagatgacaccgcgcgc			
Oex-IP*locus*_1	[2]tgttactagatcGCGGTGCTGTCCCG	*U. maydis* gDNA	964	To amplify a sequence downstream of the *IP locus* for HR.
Oex-IP*locus*_2	[3]*ttt***GAATTC**GCAACGGATTCTACGATACCTGG			
[4]oex-Verif_1	CCTGCTTGACTTGTGACCATGCC	oexUMAG_11067 gDNA	4086	To verify insertion of the overexpression cassette in *IP locus*
[4]oex-Verif_2	CGACTTTGCCTGGTGCTGAC			
[4]oex-Verif_1	CCTGCTTGACTTGTGACCATGCC	oexUMAG_11067 gDNA	1760	To verify insertion of the overexpression cassette in *IP locus*
[4]oex-Verif_3	CTGGAGCAGTTCATGATGGTAAG			
[4]oex-Verif_4	CGCTGAACAGATCCTCATTGACC	oexUMAG_11067 gDNA	1877	To verify insertion of the overexpression cassette in *IP locus*
[4]oex-Verif_5	CATCAAATCAACGTCAGCCGTCG			
q-Um11067_1	CGATGGCTTCCGCAATTA	*U. maydis* cDNA	146	To quantify gene expression of UMAG_11067 by qPCR
[5]q-Um11067_2	CGTTGTAGTTGAGACCAAGAG			
q-Um04869_1	CTTGCTACGGTCCAACATTTC	*U. maydis* cDNA	149	To quantify gene expression of UMAG_11067 by qPCR
[5]q-Um04869_2	TCGCTACTCTCCCTACTCAA			

[1] Thymine in lowercase and italics were added to increase the digestion efficiency after PCR amplification. Bold nucleotides indicate restriction recognition sequence for NcoI enzyme.

[2] The lowercase letters indicate nucleotides to fusion the PCR products by double-joint PCR.

[3] Thymine in lowercase and italics were added to increase the digestion efficiency after PCR amplification. Bold nucleotides indicate restriction recognition sequence for EcoRI enzyme.

[4] The use of this set of oligonucleotides on *U. maydis* SG200 gDNA does not produce any PCR product.

[5] These oligonucleotides were used to synthetize the first strand cDNA from RNA.
