# Supplementary material for: Enhanced oxidative stress resistance in Ustilago maydis and its implications on the virulence
Source: Int Microbiol. 2024 Feb 24;27(5):1501–11. doi: 10.1007/s10123-024-00489-8 (PMC11452521; doi:10.1007/s10123-024-00489-8)
Supplement: Supplementary file 4 — Supplementary file4 (PDF 458 KB) [file 10123_2024_489_MOESM4_ESM.pdf]

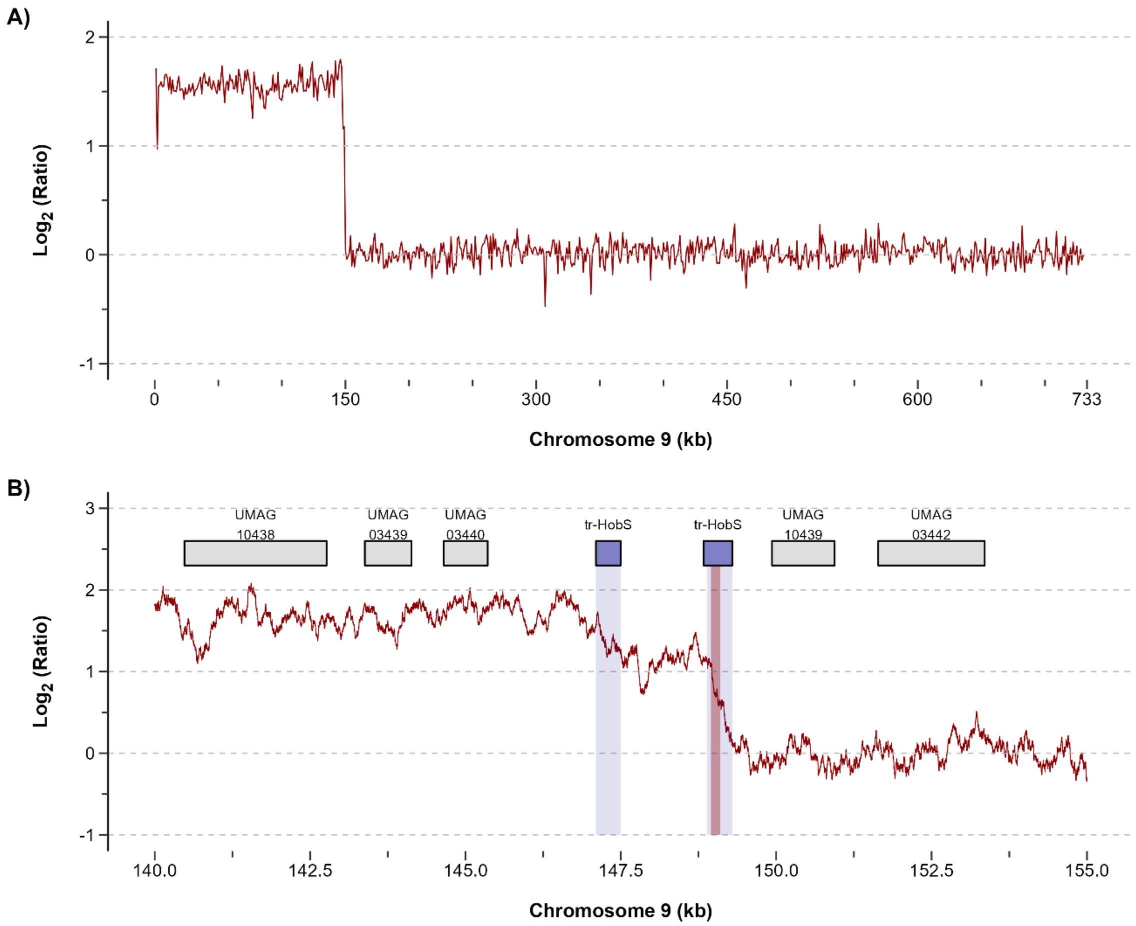


**Supplementary Figure 1. Amplification of chromosome nine. A)** $Log_2$ ratio of normalized coverage at resolution of 1 kb in non-overlapping windows in the chromosome nine in the adapted colony ($UmH_2O_2$-R Adapted Strain). **B)** Analysis at base-pair resolution of the $log_2$ ratio of the normalized coverage of the strain with amplification of chr 9.

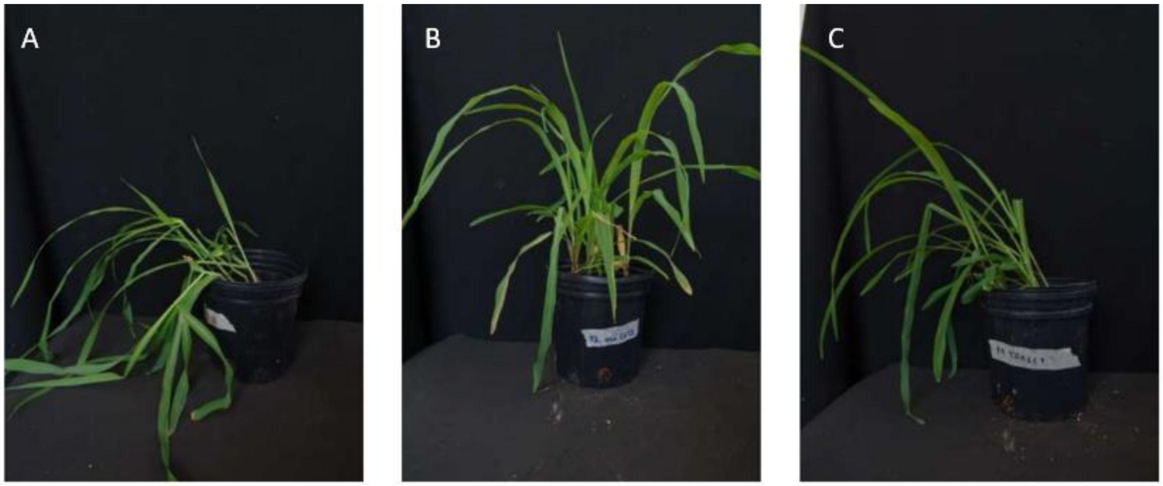


**Supplementary Figure 2. Virulence assay at 12 days-post-infection. A)** *U. maydis* SG200. **B)** oexUMAG_11067. **C)** Um$H_2O_2$-R adapted strain

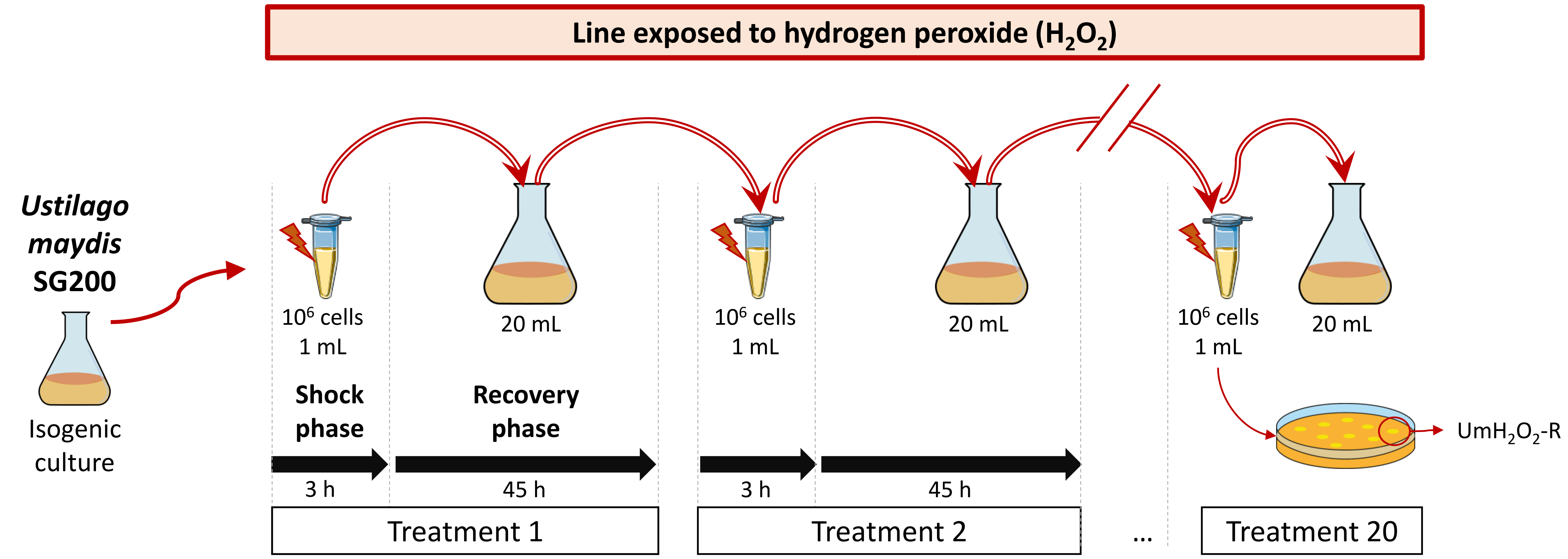


**Supplementary Figure 3.** Experimental approach to adapt the strain *U. maydis* SG200 to hydrogen peroxide. The adapted strain is denoted as $UmH_2O_2$-R after 20 intermittent shocks with arising concentrations of $H_2O_2$.
